# Design of Fly Ash-Based Alkali-Activated Mortars, Containing Waste Glass and Recycled CDW Aggregates, for Compressive Strength Optimization

**DOI:** 10.3390/ma15031204

**Published:** 2022-02-05

**Authors:** Sérgio Miraldo, Sérgio Lopes, Adelino V. Lopes, Fernando Pacheco-Torgal

**Affiliations:** 1 CEMMPRE, Department of Mechanical Engineering, University of Coimbra, 3030-788 Coimbra, Portugal; 2 INESC, Department of Civil Engineering, University of Coimbra, 3030-290 Coimbra, Portugal; avlopes@dec.uc.pt; 3 C-TAC Research Centre, Engineering School, University of Minho, 4800-058 Guimarães, Portugal; torgal@civil.uminho.pt

**Keywords:** alkali-activated mortar, fly ash, recycled aggregates, waste glass, compressive strength

## Abstract

Alkali-activated mortars and concretes have been gaining increased attention due to their potential for providing a more sustainable alternative to traditional ordinary Portland cement mixtures. In addition, the inclusion of high volumes of recycled materials in these traditional mortars and concretes has been shown to be particularly challenging. The compositions of the mixtures present in this paper were designed to make use of a hybrid alkali-activation model, as they were mostly composed of class F fly ash and calcium-rich precursors, namely, ordinary Portland cement and calcium hydroxide. Moreover, the viability of the addition of fine milled glass wastes and fine limestone powder, as a source of soluble silicates and as a filler, respectively, was also investigated. The optimization criterium for the design of fly ash-based alkali-activated mortar compositions was the maximization of both the compressive strength and environmental performance of the mortars. With this objective, two stages of optimization were conceived: one in which the inclusion of secondary precursors in ambient-cured mortar samples was implemented and, simultaneously, in which the compositions were tested for the determination of short-term compressive strength and another phase containing a deeper study on the effects of the addition of glass wastes on the compressive strength of mortar samples cured for 24 h at 80 °C and tested up to 28 days of curing. Furthermore, in both stages, the effects (on the compressive strength) of the inclusion of construction and demolition recycled aggregates were also investigated. The results show that a heat-cured fly ash-based mortar containing a 1% glass powder content (in relation to the binder weight) and a 10% replacement of natural aggregate for CDRA may display as much as a 28-day compressive strength of 31.4 MPa.

## 1. Introduction

Reducing the environmental footprint of many of the economic activities that enable human progress is a difficult task. The natural world contains delicate equilibriums, which are often incompatible with the scale and nature of human undertakings, and while humanity has been able to ignore this issue for thousands of years, the negative environmental impacts of certain activities of mankind are nowadays so pervasive that their effect can now be felt in virtually every aspect of human life. Given the scale at which raw natural resources are consumed, ecosystems are destroyed and pollution is generated by the construction sector, and despite being one of the fundamental tools of human development, this field is one of the primordial targets for intervention, when policies concerning the mitigation of the environmental impacts of human activity are designed. Two of the most efficient practices which can be implemented to improve the eco-efficiency of construction activities are, on one hand, the selection of materials produced with the help of more sustainable processes, and on the other, the adoption of the use of recycled materials. The urgent need for recycling measures has already been inscribed in the Europe 2020 Strategy for smart, sustainable and inclusive growth [1,2]. These documents stress the importance of the practical implementation of the concept of a circular economy (CE), a notion that emerged from the need to increase resource use efficiency, while minimizing resource inputs, waste and the generation of pollutants.

The vast majority of concrete compositions (one of the most used materials in the world) is produced with the help of ordinary Portland cement (OPC). Reflecting the previously stated environmental concerns, there has been a substantial effort from the scientific community to provide solutions that allow for the inclusion of higher rates of waste materials in OPC-based concretes [3]. There is currently, nevertheless, an alternative to this approach, which is to produce concrete and mortar compositions using a binder which is manufactured through a more sustainable processes and which has also been shown to be more adequately suited for accommodating higher rates of recycled materials; this family of products is called alkali-activated binders. Contrary to cement-based binders, which derive their main engineering properties from the hydration of a pozzolanic material (OPC), alkali-activated binders (AAB) are based on the alkaline activation of materials composed of alumina and silica, a much more diverse group of products, which may include recycled materials, such as fly ash and ground granulated blast furnace slag [4]. Furthermore, given that the production of cement is responsible for the overwhelming majority of the carbon dioxide emissions generated in concrete manufacture [5] and that the calcination of cement clinker (usually at temperatures superior to 1400 °C) involves high energy use and the generation of CO_2_ (as a by-product of the decomposition of limestone), the fact that the utilization of OPC may totally be avoided represents one additional advantage of this family of products. Nevertheless, the evaluation of the environmental impacts of the use of AAB in concrete and mortar compositions is still ongoing, and even in 2013, an investigation stated that, while the environmental footprint of geopolymers is reported to be highly correlated with the alkaline activators’ production process and with the potential heat curing regimens implemented, the carbon dioxide equivalent of geopolymer concrete is only 9% inferior to that of an equivalent OPC concrete [6]. However, more recent studies suggest that, for the compressive strength resistance class, the CO_2_ emissions associated with the production of fly ash-based geopolymers are about 63% (or 166.36 kg CO_2_/m^3^) lower than the ones generated in the production of an OPC-based concrete and that there is no significant increase in emissions by promoting the upgrade in compressive strength [7]. Moreover, the scientific literature also suggests these materials have superior performance in resisting acid attack [8] and freeze-thaw cycles [9], lower susceptibility to the occurrence of the alkali-silica reaction when reactive aggregates are present [10] and higher capacity for the immobilization of wastes [11], when compared to OPC-based compositions.

One of the wastes which has been thoroughly used as a precursor in alkali-activated binder mortars and concretes both in scientific literature and practical applications is fly ash [12]. This material is a by-product of the coal-fired power generation process, as it results from the electrostatic precipitation of the burned fuel particles that are driven out of coal-fired boilers (activity which, in itself, also leads to the generation of flue gases). Despite global efforts to reduce the production of energy from non-renewable sources, coal ash, according to a 2014 estimate [13], was still being generated in coal-fired power stations at a rate of around 130 × 10^6^ t per annum. This by-product is a fine granular aluminosilicate, which displays low pozzolanicity and possesses a low calcium content, although when activated with an alkaline solution (typically a combination of sodium hydroxide and sodium silicate), it forms a binder in which the main reaction product is an amorphous alkaline aluminosilicate hydrate known as N-A-S-H gel, which is mainly responsible for the development of its mechanical strength [14]. One of the peculiarities of cementitious products based on the alkaline activation of fly ash is the fact that, in order to display high compressive strength, the curing of the specimens must be performed with the aid of a high temperature and/or humidity process [14], which invalidates a large number of applications for these products. Nevertheless, 24-h curing regimens at temperatures in the range of 60–90 °C and also the utilization of NaOH concentrations with values situated above 14 M have been shown to produce fly ash-based mortars possessing compressive strengths superior to 100 MPa, measured immediately after the heat curing stage ends [15]. In addition, as described in [16], research shows that alkali-activated concretes, including fly ash-based compositions, have showed equivalent performance to OPC-based concretes in structural applications.

However, the selection of the binders, and therefore production processes, for mortars and concretes, is not limited to a choice between traditional OPC-based mixtures, and alkali-activated compositions. In fact, there is a class of binders, termed “Portland-alkaline cements”, which has been receiving growing attention from the scientific community. These binders contain blends of OPC and aluminosilicates (such as fly ash and/or blast furnace slag) and, by reducing the cement content of the mixtures, allow for the upgrade of the environmental performance of mortar or concrete compositions. In addition, this process also enables the possibility of using higher amounts of non-calcined clays (such as bentonite [17,18]). The vast majority of the mortar compositions designed and produced, in this work, can be classified, due to their high OPC content, as hybrid alkali-activated mortars. The chemical mechanisms behind the activation of hybrid alkali-activated mortars are of substantial complexity and are still poorly understood [14,19], in fact, a typical challenge observed in these compositions is the initial rapid development of compressive strength after the high temperature curing period, and the degradation of this strength with the evolution of curing time [20]. Consequently, it becomes clear that this field is in need of higher research attention.

The reduction of the sodium silicate content of an AAB composition is a measure which further improves the environmental sustainability of its fabrication as its production process is highly energy intensive and utilizes a great number of natural resources [6]. However, as this product’s main function is to provide soluble silicates to the alkaline solution, the strength performance of the mixtures will consequently be negatively impacted by this reduction. In this investigation, a mitigation measure for the reduction sodium silicate content was studied. This method consists of providing soluble silicates to the mixture by balancing the silicate decrease with the addition of fine recycled waste glass powder.

One of the main advantages of the use of glass wastes is its abundance. In the EU, for example, and according to the EUROSTAT online database on waste generation [21], in 2018 alone, the amount of glass wastes generated by the 27 members was of 16.37 × 10^6^ t. The main justifications behind the applicability of the use of the finely milled version of this silica-rich product in cementitious compositions is that it has been shown that it may be used, on one hand, to produce viable sodium silicate solutions for use in AAB compositions [22,23,24,25]. Furthermore, it has also been shown that this product may be effective, as long as the expansions related to the alkali-silica reaction are controlled in partially replacing cement as a pozzolanic material in the production of mortars and concretes [26].

As already mentioned, the analysis of the properties of AAB-based mortars and concretes suggests that, when compared to OPC-based mixtures, these materials possess a higher tolerance for the incorporation of, on the one hand, higher rates, and on the other hand, a wider diversity of wastes [27]. This capacity demonstrated by AAB-based materials is demonstrated by the fact that, contrary to OPC-based mortars and concretes, capable concretes can be produced with the help a high volume of fly ash or ground granulated blast furnace slag (in fact, the precursors can be solely composed of such wastes). Additionally, AAB-based materials demonstrate a higher capacity for the immobilization of harmful wastes, and also, due to the fact that the compressive strength of geopolymers is not as dependent upon the aggregates’ particle packing optimization, it rather strongly relies on the characteristics of the geopolymer matrix [28].

One of the applications of AAB-based materials which demonstrates greater potential is the case of the utilization aggregates sourced from construction and demolition waste (CDW) in concrete compositions. According to the previously mentioned EUROSTAT dataset, which concerns the total waste generation of EU-27 countries in 2018 [21], mineral waste from CDW production for the same year and region was almost 300 × 10^6^ t (or approximately 13% of total waste produced). Traditionally, one of the most important obstacles to the use of these products in concrete compositions is that, depending on the properties of the original materials, their state of decay and the removal/demolition approach selected to recycle them and also the quality and type of materials which can be obtained from recycling CDW is highly variable [29]. A major factor contributing to the quality of concrete containing recycled CDW aggregates is the microstructure of the region situated between the aggregate and the cement paste, the interfacial transition zone (ITZ), as it is critical to the sound development of the mechanical properties of the concrete. When coarse aggregates composed mainly of recycled concrete (here designated as construction and demolition recycled aggregates—CDRA) are used in OPC-based concretes, and due mainly to the presence of old adhered paste, several ITZs are created (thus multiplying the occurrence of weaker regions in the concrete). However, analysis of the ITZ has showed that when CDRA are used in AAB-based concretes, the new binding material “has the ability to reach the incomplete interphase and create geopolymerization-hydration products” and thus produce a concrete with higher mechanical properties [30].

Another well-known strategy for improving the microstructure of mortars or concretes is the inclusion of a filler. Once again, this presents an additional opportunity for the inclusion of recycled wastes in the mixture. Several waste materials have already been shown to adequately improve the performance of OPC-based concretes [3] and there is also some evidence that this addition may also be beneficial, although to a lesser extent, in alkali-activated compositions [31]. In this investigation, the effects of including limestone waste powder as filler in mortar compositions were studied.

The present work focuses on the utilization of a fine waste that can be sourced from commercial CDW recycling plants (amongst others) and which is mainly composed, not only of masonry or cementitious products, but also of materials like wood, plastic or asphalt, among others. Although an official designation for these recycled aggregates does not exist, in this investigation, and similarly to other existing literature on this subject [32], this waste will be designated as construction and demolition recycled aggregates (CDRA). The properties of this waste are highly variable and the practical application of this material in cementitious materials is highly restricted. Such is the case of Portugal, for instance, where its National Laboratory for Civil Engineering (LNEC) prohibits the use of fine CDRA in concrete [33] and, on the other hand, restricts the utilization of the coarse fraction of the waste in concrete compositions to a maximum of 20–25% of the total aggregate weight. Nevertheless, recent studies [34,35] suggest that small additions (ideally, 0.3–0.4% of binder weight) of super absorbent polymers (SAP) in OPC-based self-compacting concretes in which up to 15% of the cement weight is replaced by non-biodegradable granite pulver, while inducing a loss in the workability of the mixtures, leads to improvement in the mechanical strength of the compositions (compressive, splitting tensile and flexural strengths) while reducing the weight loss due to acid attack (although the strength displayed by the concrete is impacted by the addition of the SAP).

In summary, the experimental work carried out consists of the development of a methodology to elaborate an eco-efficient mixture displaying adequate compressive strength. The simultaneous utilization of precursors composed of fly ash, Portland cement, calcium hydroxide and fine milled waste glass is a new approach, in a field (hybrid cements) where literature is scarce. Moreover, the study of the inclusion of fine CDRA in the mortars is also important to access the potential of geopolymers to accommodate higher waste contents.

The research work was performed in two major phases, one in which the object of investigation was the short-term compressive strength of ambient-cured mortar samples and another in which the investigation focused on the determination of the 28-day compressive strength of heat-cured mortar samples. For this purpose, several 40 × 40 × 160 mm parallelepiped samples were produced and subsequently submitted to compression tests [36]. As can be seen below, recycled sand and waste glass constitute the residues to be incorporated into the mixtures (along with, to a lesser extent, waste limestone powder).

## 2. Materials and Methods

### 2.1. Materials

#### 2.1.1. Precursors

The primary precursor of the mixtures which can be found in this work is low calcium (type F) fly ash. This material was originally sourced from a Portuguese coal-fired power plant in Sines, CIMPOR, which afterwards tested and supplied the material to this investigation. According to the testing performed, this material fulfills the requirements stated in the European standard concerning fly ash use in concrete applications [37]. The main characteristics (such as chemical composition, reactive silica content and fineness) of the fly ash used in this study can be seen in Table 1.

Another highly important material thoroughly used in this investigation is ordinary Portland cement. The presence of OPC as starter material in an AAB concrete, as previously mentioned, allows for the simultaneous generation of supplementary calcium-based gels and therefore aids in the development of the mechanical strength of the mixture. In this investigation, the authors made use of type I 42.5 R Portland cement, most of which was sourced from the CIMPOR production facility in Souselas, Coimbra, Portugal.

Furthermore, the addition of slaked lime (calcium hydroxide) was also evaluated as a further source of calcium and as an accelerator of the setting of the samples.

#### 2.1.2. Waste Glass

The viability of using glass wastes as a source of silica in alkali-activated mortars for the improvement of mechanical strength results was analyzed through the addition of crushed used soda-lime bottles up to a particle size of less than 500 µm. The main objective of this procedure was to provide soluble silicates to the binder, with the intention of reducing the sodium silicate content of the mortar and therefore enhancing its eco-efficiency.

**Figure 1:**
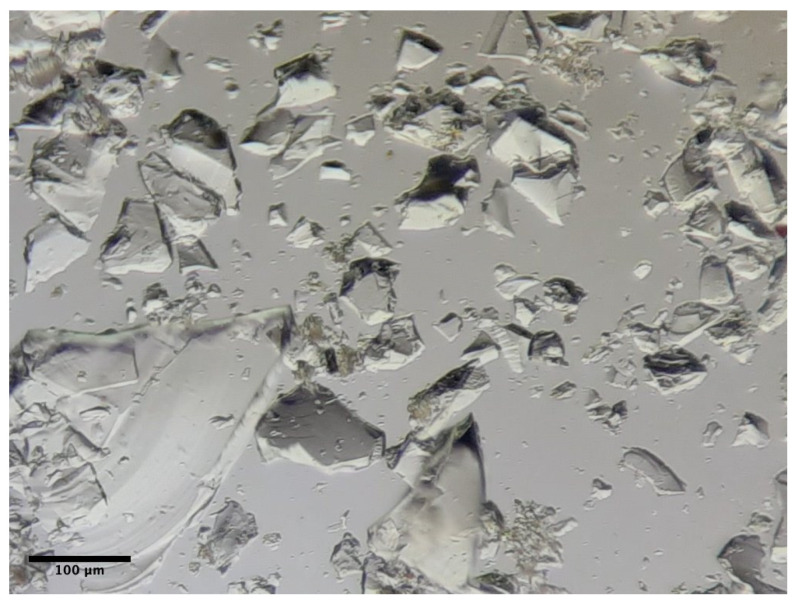
Crushed soda-lime bottles glass (10× magnification; Φ < 500 µm).

As to the production process of the powder, before reaching the crushing stage, the bottles were thoroughly washed, and all the non-glass materials were removed. The crushing process was performed via the insertion of the bottles and the steel balls in a Los Angeles abrasion test machine and then performing standard abrasion test cycles of 500 revolutions at a speed of 31 revolutions per minute [38]. Afterwards, the residue was sieved to obtain glass particles dimensionally inferior to the desired size. An optical microscope image of a sample of the crushed waste glass used in this study, in which the shape of the crushed glass particles can be observed, is presented in Figure 1.

To produce sodium hydroxide solutions, and in order to reduce the probability of the presence of impurities, pellets with 98–99% purity were dissolved in distilled water until the target concentration was reached.

As to the other alkaline activator used in this work, sodium silicate, its chemical composition is indicated in Table 2.

#### 2.1.3. Aggregates

**Figure 2:**
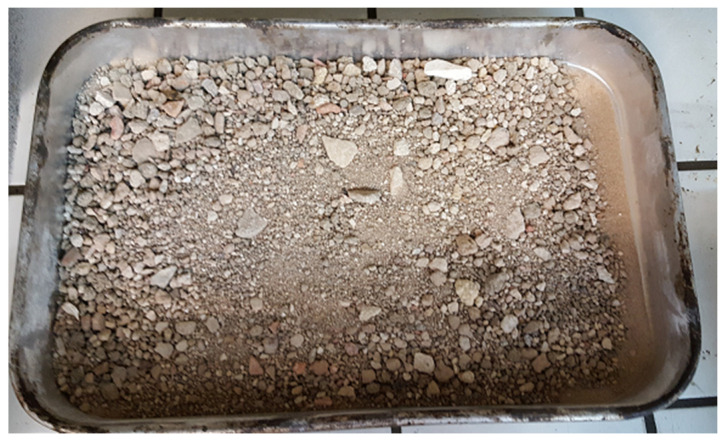
Dried construction and demolition recycled aggregates.

The fine recycled aggregates used in this work (Figure 2) were obtained from an operating commercial CDW recycling plant located in Figueira da Foz (RCD—Resíduos de Construção e Demolição). As to the recycling process implemented in this facility, prior to being introduced in the production line of the industrial plant, the raw CDW is not subjected to any sorting. The fine material resulting from the physical processes used by this company to separate the vast majority of the waste’s contaminants is a somewhat heterogeneous aggregate which possesses a particle size within the 0/10 mm range and which is rarely recycled into OPC-based concretes.

**Figure 3:**
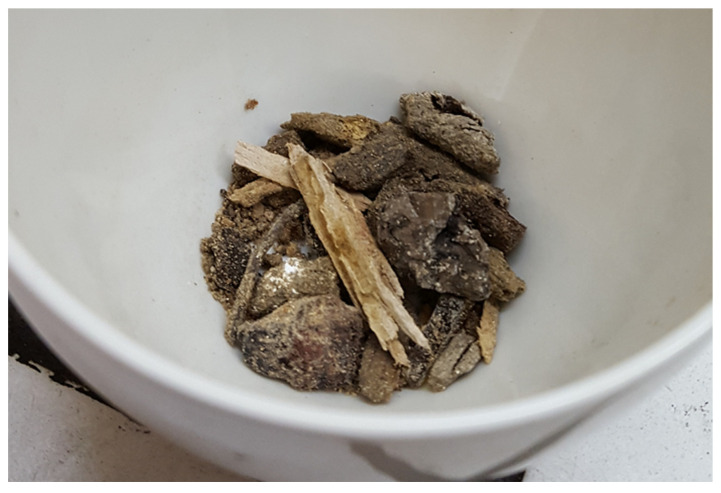
Example of contaminants found in a random sample of CDRA.

To further illustrate this reality, an image of the contaminants that it was possible to remove from a sample of dried fine recycled aggregates after performing a visual inspection is presented in Figure 3.

Previously to being transported to the laboratory, the recycled aggregates in the plant were stored unsheltered in the plant. For this reason, to remove the humidity, the aggregates were dried at a temperature of 110 °C to constant mass and then sieved to obtain the fraction 0/8 mm for use in the production of the mortars.

**Figure 4:**
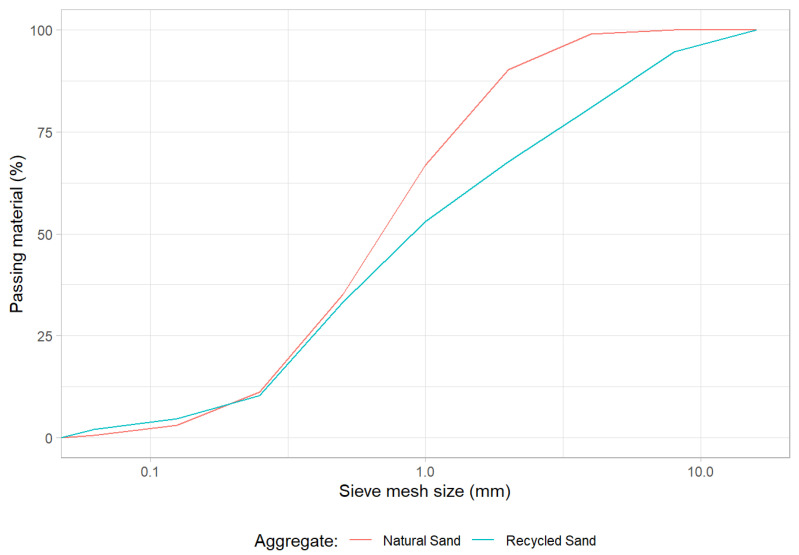
Grading of the natural and recycled aggregates.

In addition, sand of the fraction 0/4 mm was used as fine natural aggregate in the production of the mortars. The particle size distribution of both aggregates can be observed in Figure 4.

Finally, it is important to mention that, in specific compositions, the upgrade of the aggregates particle size distribution was also evaluated through the use of a filler composed of waste limestone powder.

#### 2.1.4. Water-Reducing Agent

A high-performance superplasticizer based on modified polycarboxylates, Sika ViscoCrete 3005 (supplied by Sika Portugal), was added to the mixtures in order to reduce the hydration needs of the mortars (especially of the recycled aggregates) and thus optimize the particle packing of the mortars.

### 2.2. Mix Design

The current section details the design principles subjacent to the conception of the eco-efficient mortars produced in the scope of this work. Also presented are the detailed material compositions of the mentioned mixtures.

This investigation was preceded by a long stage of preliminary compressive strength testing performed on experimental samples. In this stage, several factors, such as the optimal curing temperature, recycled aggregate replacement rate and several material proportions were established and thus this work presents only the fine tuning of the optimization of the design of mixtures and the compositions and not the justification for every design decision previously taken.

#### 2.2.1. Initial Testing Stage

The main goal of the first testing campaign was the maximization of the compressive strength of ambient cured fly ash-based alkali-activated mortars containing waste glass and recycled aggregates. For this purpose, an initial set of three mortar mixtures was designed and the main material properties of these mixtures can be observed in Table 3.

As can be observed, the majority of the binder’s weight was, in all mixtures, composed of fly ash. Moreover, ordinary Portland cement, slaked lime and waste glass powder were used as secondary precursors in an attempt at maximizing the compressive strength of the samples. Furthermore, following the knowledge acquired during the already mentioned preliminary testing stage (and to current available knowledge), 10% of the natural aggregates were replaced with CDRA. This replacement typically hinders the workability of the mixtures, and thus, the use of a superplasticizer was implemented (in a proportion of 1%, relative to binder weight). The remaining material ratios of the compositions, such as activator to binder ratio, sodium silicate to hydroxide ratio, hydroxide concentration and aggregate to binder ratio are situated within the usual range of fly ash-based geopolymers.

Following the first round of compression tests, the investigation focused on analysis of the effects of increasing the OPC content of the mixes on the compressive strength of the mortars and simultaneously the consequences of reducing the hydroxide concentration (from 16 M to 10 M) and reducing the activator to binder ratio (from 0.7 to 0.6) in mixtures in which the binder was composed of fly ash and OPC in equal parts (Table 4).

In the third step of the initial compressive strength optimization stage, the most important modification of the mortar design was the substantial increase of the silicate to hydroxide ratio (from 1 to 2). In addition, for one of the mixtures (MM8), the alkaline solution to binder ratio was reduced, while simultaneously, the CDRA were completely replaced by natural aggregates (Table 5).

#### 2.2.2. Waste Glass Content Optimization Stage

Building on the results provided by the previous stage, a new phase of the work was devised. More specifically, it was deemed important to investigate the strength performance of heat-cured compositions as the inclusion of a heat-curing stage would allow for compositions containing lower cement contents and to potentially reduce the silicate content of the alkaline solution. This strategy also allowed for the study, in more detail, of the influence of the addition of waste glass powder on the mechanical performance of the mortars.

As the addition of the glass waste implies the modification of the remaining material proportions, it was clear that a methodology would have to be devised in order to minimize the impact of the other material relationships on the final compressive strength. Consequently, and in light of the data provided by the initial testing stage, a reference composition was designed. This mortar was designed so that oven cured samples would achieve the highest compressive strengths possible, while minimizing the samples’ OPC content. The composition of the reference sample can be seen in Table 6.

After designing the reference sample, two sets of mixtures based on variations of this composition were formulated:

1. one set in which the addition of increasing amounts of waste glass (namely, 1%, 4% and 7% of the weight of the precursors) was compensated by a reduction in sodium silicate (plus proportionate adjustments in the fly ash, cement and aggregates content in order to maintain overall weight proportions);
2. another group in which the same waste glass additions were matched by the increase in sodium hydroxide and decrease in sodium silicate (in this case, only cement and fly ash contents were impacted by the waste addition).

The variation in the relative mass (in relation to the total weight of the mixture) of each component of the mixtures designed according to the previously stated principles (group one and two of this section) is shown in Table 7.

For further clarification, the most important material ratios of the compositions resulting from the application of the first and second approach of the described method, respectively, are presented in Table 8 and Table 9.

### 2.3. Specimen Preparation

All compositions were produced according to the specifications stated in the EN 196-1 standard [39]. More specifically, the binder’s components (cement, fly ash, calcium hydroxide and glass) were first mixed together for one minute in a mortar mixer. The aggregates were then added to the binder, and the resulting mixture was also blended for one minute. Meanwhile, the formerly dissolved sodium hydroxide was thoroughly mixed with sodium silicate in a plastic container, allowed to react, and then added to the binder and aggregates. At this stage, if the addition was contemplated in the design of the mortar, the superplasticizer was then added to the mixture.

The resulting mortar was poured, in two equal layers, into 40 × 40 × 160 mm steel moulds. After the first layer was poured, a vibrating table was used to remove the air from inside the mixture. Then, the mold was filled by pouring the last layer of material and repeating the vibration process. Afterwards, the specimens were wrapped in plastic film and allowed to cure, either at room temperature or at 80 °C, for twenty-four hours, in an electric oven (methodology also followed by [7,40] and within the range of temperatures and curing periods usually adopted in the literature), after which period, the specimens were de-molded and covered in film plastic and left at room temperature, until being tested for compressive strength at the target curing age.

No extra water was used in the production of the mortars.

### 2.4. Test Methodology

**Figure 5:**
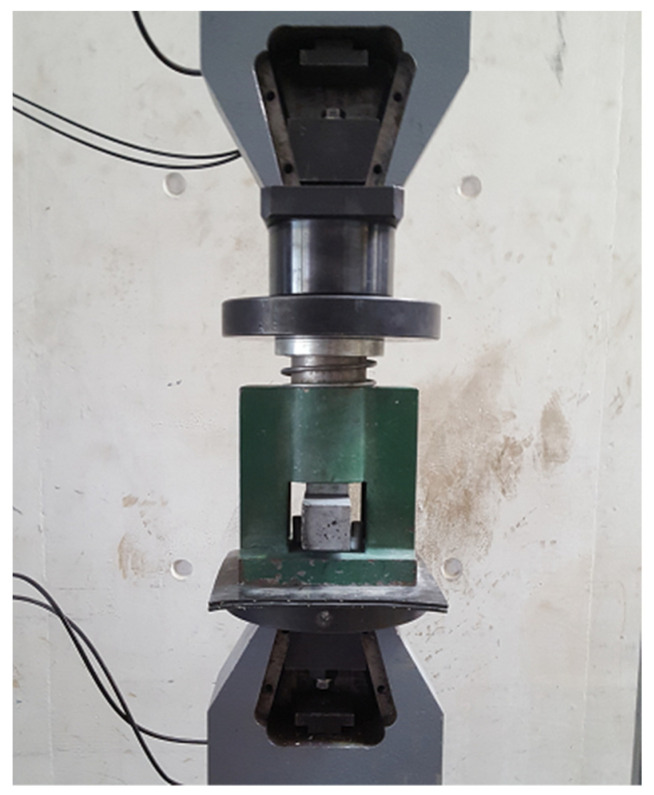
Compressive strength test set-up.

Each 40 × 40 × 160 mm sample allowed for the compressive strength test to be performed three times, as the test machine was equipped with an adapter which transferred the loading from the plates to a 40 × 40 mm square. For logistical reasons, in each of the two stages of optimization, two different load testing machines were used (although the exact same procedures were used in both). The compressive strength test set-up used in the first stage of optimization is presented in Figure 5.

**Figure 6:**
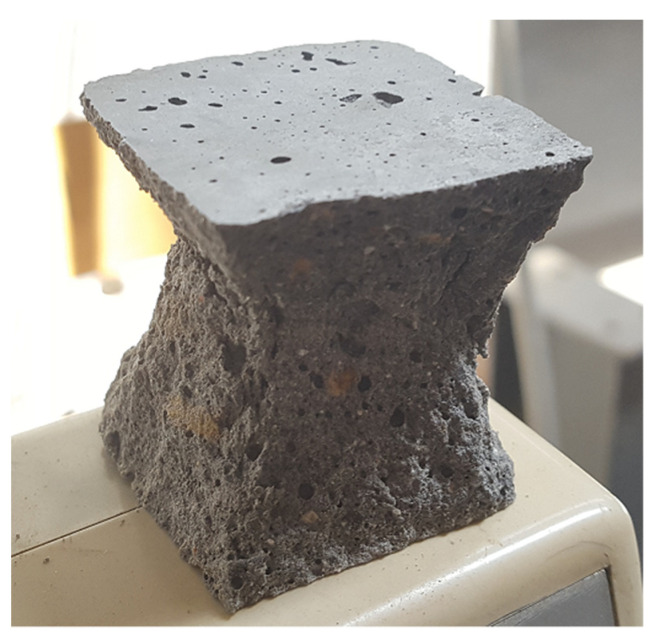
Fly ash-based AAB mortar specimen displaying conical semi-explosive failure.

For each mixture, three cubic 40 mm × 40 mm × 40 mm samples were tested. The specimens were, as required by the European standard [36], taken to conical semi-explosive failure (Figure 6), starting at a loading rate of 0.1 mm/s, up to about 10% of maximum load, and secondly at a rate of 0.013 mm/s. The resulting strength versus displacement data was then analyzed.

## 3. Results and Discussion

### 3.1. Initial Testing Stage

**Figure 7:**
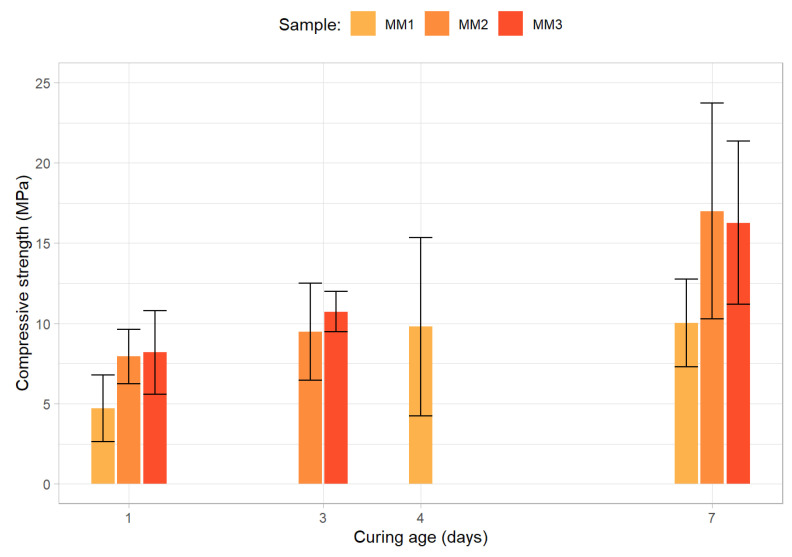
Compressive strength results for the first round of the initial optimization stage.

As previously mentioned, the ten mixtures that belong to the initial stage were all tested up to 7 days. The average compressive strength for each of the curing periods and for each of the samples belonging to the first group of mortars, along with the 95% confidence interval for each sample and curing period (represented by the error bars), are presented in Figure 7.

As can be observed, even when 40 to 50% of the precursor’s fly ash content is replaced by secondary precursor materials and, simultaneously, a high NaOH concentration (16 M) is utilized, the maximum average 7-day compressive strength obtained by the ambient cured mortars is inferior to 18 MPa. In addition, the utilization of a 7.5% cement content (in relation to the weight of the binder) results in a mortar displaying significantly lower average 7-day compressive strength (around 10 MPa).

**Figure 8:**
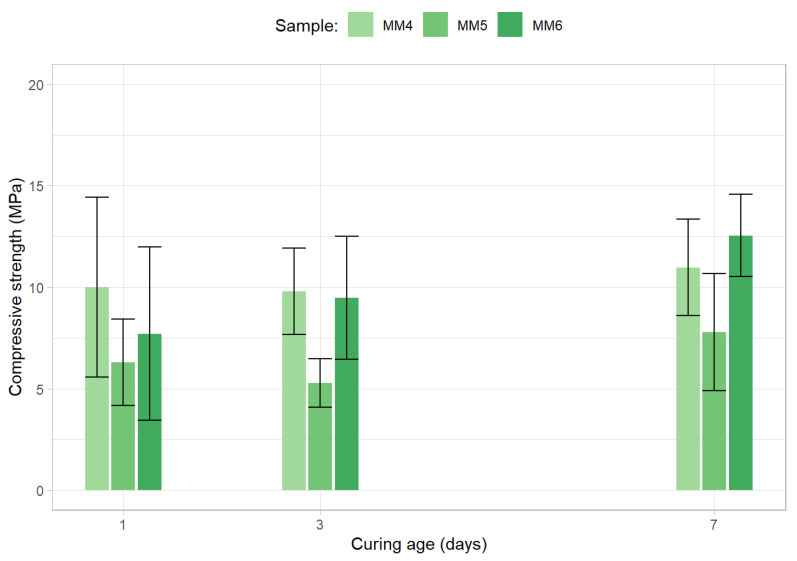
Compressive strength results for the second round of the initial optimization stage.

The investigation now focused on mixtures containing a higher OPC content (50%) but lower silicate to hydroxide ratio (1). The compressive strength results can be seen in Figure 8.

The deterioration in strength results demonstrated by the compositions belonging to the second round of tests, in which every mixture has a low silicate to hydroxide ratio (1), in comparison to the strengths displayed by the first group of mortars show the importance of this ratio for the development of compressive strength in alkali-activated materials. In fact, despite possessing high OPC content, the best sample of the group displayed an average 7-day strength of only 12.5 MPa. Moreover, it can be observed via the analysis of the compressive strength values of MM4 and MM5 that, contrary to what would be expected, the increase of the sodium hydroxide concentration has a negative impact on the strength displayed by the samples. This phenomenon may be explained by the fact that the increase in hydroxide concentration leads to a decrease in the water content of the samples and thus to a lack of a minimum water threshold for achieving a proper cement hydration process (especially when considering that recycled aggregates require more water than their natural counterparts and that the samples’ binder cement content was 50%). Furthermore, the data from the MM6 sample test suggests that the decrease in alkaline solution (0.7 to 0.6 activator to binder ratio reduction, in relation to MM4), and therefore in solid alkaline activators, is not as important for strength development, in these conditions, as the decrease in the liquid phase of the activator.

**Figure 9:**
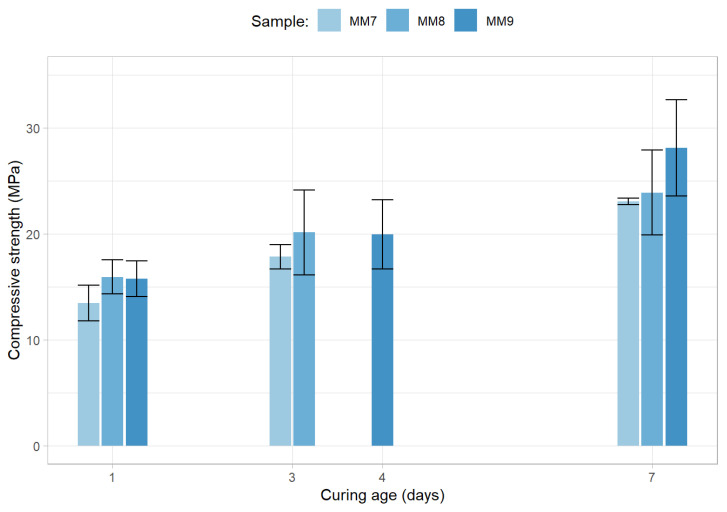
Compressive strength results for the third round of the initial optimization stage.

Finally, Figure 9 contains the strength results from the third round of mixes.

The results provided by these compositions show the positive effects of increasing the silicate to hydroxide ratio (from 1 to 2) as the range of the 7-day average compressive strength was the highest achieved thus far (in fact, even the worst-performing composition—MM7—displayed a strength of 23.1 MPa). In addition, similarly to what was observed in the previous round of testing, the reduction in alkaline solution did not lead to a reduction in strength (although a direct comparison between MM7 and MM8 cannot be made, as the recycled aggregates and limestone powder rate are also different in both mixtures). Nevertheless, the results clearly indicate that limestone powder additions are beneficial to the strength of mixtures. In reality, mixture MM9, which contains a 10% limestone powder for natural aggregates replacement, demonstrated a 7-day compressive strength of 28.1 MPa.

The strength results obtained in this stage of the investigation partly agree with the ones reported by [41], in which they studied the influence of OPC additions on, amongst other properties, the compressive strength of ambient-cured low-calcium fly ash mortars. In this study, the authors state that the ambient-cured mortar, in which the precursor was composed only of fly ash, displayed negligible 3-day compressive strength, while the addition of a 5% cement addition (in relation to the precursor’s weight) caused an improvement of the 3-day compressive strength to 13 MPa. Nevertheless, these researchers also report a 7-day compressive strength for the mortars containing 5% cement content of over 25 MPa, which is considerably higher than the 10 MPa strength achieved by this investigation for the mixture containing 7.5% cement (MM1). In fact, only a substantial rise in cement content (up to 50% of the precursor weight) led to 7-day compressive strength values situated in the same range as the results presented in the mentioned investigation (23 MPa of this work’s MM7 versus the already mentioned 25 MPa stated in the literature).

The disparity between the outcomes of both investigations is likely predominantly due to the difference in the comparable compositions of the mortars present in each investigation, namely, the recycled aggregates content (10% vs. 0%), lower sodium hydroxide concentrations (10 vs. 14M) and lower silicate to hydroxide ratio (2 vs. 2.5) used in the composition.

Furthermore, chemical, physical and microstructural differences of the fly ash used in each study may have also played a role in the contrasting strength results. In particular, the low value of the Blaine specific surface area of the fly ash utilized in the present work (257 m^2^/kg) may have played a major role in the development of early-stage compressive strength of the mortars produced. In fact, recent evidence [42] demonstrates that there is a strong linear correlation (R^2^ > 0.75) between this property and the early compressive strength of fly ash pastes in up to seven days of curing (the correlation can also be improved if the Blaine specific surface area is adjusted by making use of a ‘shape factor’, which is determined through calculation of the specific surface area directly from the particle size distribution of the fly ash, and assuming that the material is composed of discrete spheres). Moreover, as mentioned in [14], the ideal content range for particles smaller than 45 µm in fly ash meant for alkali activation is 80–90%, which is considerably higher than the 69% content present in the fly ash used in the present investigation.

### 3.2. Waste Glass Content Optimization Stage

**Figure 10:**
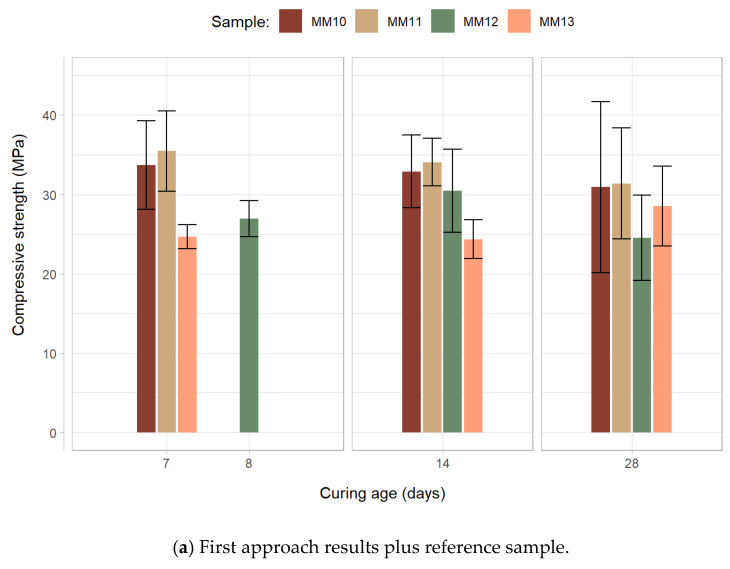

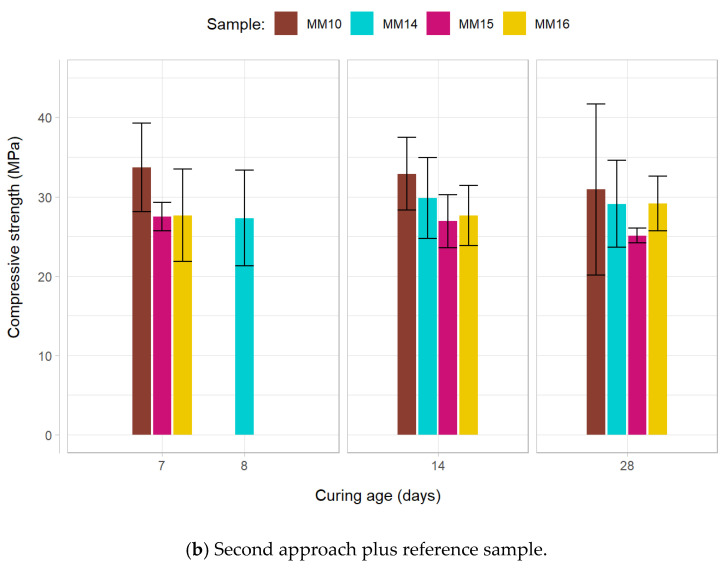
Compressive strength results for the waste glass optimization stage. (**a**) First and (**b**) second approach.

The optimization of the waste glass content was performed through a set of mixtures in which mortars containing a wide range of material ratios were tested to determine the compressive strength. As previously detailed, the addition of the increased waste glass content of the mixtures was partly accommodated by either reducing the sodium silicate content while maintaining the sodium hydroxide amount and increasing the aggregates content or by also reducing sodium silicate but maintaining the aggregates and increasing the sodium hydroxide quantities (while keeping the remaining material proportions unchanged). This methodology was designed for the assessment of which of the alternatives would be most effective in minimizing the impacts of the replacement of the mixtures’ binding materials (mostly fly ash) for glass wastes on the compressive strength of the compositions. The compressive strength results displayed by the mortars, for each of the two designed approaches, are presented in Figure 10.

As can clearly be observed via the analysis of the results, the modifications implemented for the composition of the reference sample were, with one exception, detrimental to the compressive strength of the mortars. These findings suggest that, for both approaches, the negative impacts of the cement/silicate reduction could not, with one exception, be offset by the changes promoted to the other components’ contents. Notwithstanding, results from MM11 show that that a reduction in sodium silicate (−1% of the composition weight), fly ash (−0.56%) and cement (−0.19%) may, in these conditions, be compensated by the addition of a 1% waste glass content (and a 0.75% increase in the mixture’s aggregate content). This composition displayed a 28-day compressive strength of 31.4 MPa.

To determine whether the factor ‘glass addition’ had a statistically significant effect on the ‘compressive strength’ variable, a one-way ANOVA test was conducted for each mean compressive strength and curing age period (short, medium and long term), the resulting *p*-values can be observed in Table 10.

The above-mentioned p-value results show, although for the short and medium-term curing age, a statistically significant relation between the glass addition factor and the compressive strength, as both *p*-values are situated below 0.05 (95% confidence level); the contrary can be stated in relation to the compressive strength values at 28 days, as the respective *p*-value (0.0309) suggests a statistically significant difference in the 28-day compressive strength with the increase in the mortars’ waste glass content.

The range of compressive strength results demonstrated by the mortars is superior to the ones provided by the literature. As an example, [43] obtained a 7-day compressive strength of 21 MPa when testing mortar samples composed of class C fly ash and calcium hydroxide cured for two days at a temperature of 60 °C, having observed no meaningful compressive strength increase in a period up not 28 days.

Another important conclusion that can be drawn from the analysis of the data is that the compressive strength development of heat-cured fly ash-based hybrid geopolymer (in which the activation model is based both on the activation of an alkaline solution and cement hydration) is highly unstable. In fact, the strength results are highly variable, with elevated dispersion of values both amongst the samples of the same mixtures and also for different time periods. As an example of this phenomenon, it is to be noted that the results frequently show [43,44] a decrease in strength as curing time progresses.

The explanation for this lack of stability in the strength development of the mortars is likely related to the complex chemical interactions (and competition) between the different types of gels which are typically formed in the activation of hybrid systems, as the utilization of high-calcium components, such as OPC or slaked lime, leads to the formation of mixed (C,N)-A-S-H or N-(C)-A-S-H-type gels [14,45].

In relation to the environmental sustainability gains that these mixtures may offer, the evaluation can be performed in two distinct planes. From the standpoint of the reutilization of waste materials, the superiority of this mixture in comparison to OPC mortars is evident, as it allows for the recycling of, on one hand, glass wastes and, on the other, fine recycled aggregates from CDW, a product for which there is a lot of applicability in real-world industrial applications [32]. As to the analysis of the compositions in terms of their global warming potential, according to the available literature [46], and this property’s impact factors for the constituents of the mortars, in comparison to typical equivalent OPC-based mortars, all suggest that these mixtures do not display a meaningful advantage in relation to equivalent OPC mortars in relation to their global warming potential.

## 4. Conclusions

This investigation focused on the design optimization of fly ash-based AAB mortars, based on the compressive strength performance of these mixtures. The study can be divided into two distinct stages: the first, in which ambient-cured compositions were taken to compressive strength failure after short curing periods (up to 7 days), and a second stage, conceived for testing heat-cured samples, tested at up to 28 days. The ultimate goal of the investigation was to optimize the eco-efficient performance of the compositions while simultaneously maximizing the compressive strength of the samples. To achieve this objective, the main priority of the first stage of the study was to avoid the energy intensive heat-curing period. Afterwards, the experimental work focused on the minimization of the cement content of the mixtures through the addition of waste glass powder. The most important conclusions drawn by this study are as follows:

1. It is possible to obtain 7-day compressive strength values of about 28 MPa using ambient-cured fly ash-based mortars. Nevertheless, in the case ordinary Portland cement is used, large amounts of this material (50% of the binder) and alkaline activators must be used to produce mixtures with moderate potential for structural use.
2. The implementation of a 24-h cycle of heat curing at a temperature of 80 °C allows an improved strength performance to be obtained with lower cement contents (below 25%).
3. The utilization of glass waste powder revealed some potential in offsetting a decrease in the content of the most carbon intensive products of the mixtures (sodium silicate and cement). In fact, the mixture with the best 28-day compressive strength results (M11) possessed a 1% glass powder content (in relation to the binder weight) and displayed a 28-day compressive strength of 31.4 MPa.
4. Although superplasticizers were used, the highest compressive strength results pertained to compositions with low workability.
5. In most samples, the strength results of the fly ash-based mortars displayed high dispersion, and wide 95% confidence intervals were observed. This phenomenon was more prominent in heat-cured samples.
6. A one-way ANOVA test showed that, for a 28-day curing period, the addition of glass wastes had a statistically significant effect on compressive strength.

## Figures and Tables

**Table 1:** Main fly ash properties.

Element	Content (wt.%)
SiO_2_	61.7
Al_2_O_3_	18.64
Fe_2_O_3_	6.81
CaO	1.45
Others	5.94
LOI	5.46
Reactive Silica	39.22
Part Diam. < 45 µm	62.9
Blaine Specific Surface (m^2^/kg)	257.0

**Table 2:** Chemical composition of the sodium silicate solution.

SiO_2_ (wt.%)	Na_2_O (wt.%)	Al_2_O_3_ (wt.%)	H_2_O (wt.%)
27.3–28.3	8.2–8.6	<4.0	59.1–64.5

**Table 3:** Key material ratios of the first group of mortar mixtures.

Sample	FA ^1^ (%)	CM ^2^ (%)	SL ^3^ (%)	Gl ^4^ (%)	S/H ^5^	H ^6^ (M)	A/B ^7^	RA ^8^ (%)	LP ^9^ (%)	Ag:B ^10^	Sp/B ^11^ (%)
MM1	60	7.5	12.5	20	2	10	0.7	10	10	3	1
MM2	60	25	15	0	2	16	0.7	10	10	3	1
MM3	50	25	15	10	2	16	0.7	10	10	3	1

^1^ Fly ash to binder content; ^2^ cement to binder ct.; ^3^ slaked lime to binder ct; ^4^ glass powder to binder ct.; ^5^ sodium sil. to hyd. ratio; ^6^ sodium hyd. concentration; ^7^ alkaline sol. to binder rt.; ^8^ recycled to total aggr. rt.; ^9^ limest. pow. to total aggrrt.; ^10^ aggregates to binder ratio; ^11^ superplast. to binder rt.

**Table 4:** Key material ratios of the second group of mortar mixtures.

Sample	FA ^1^ (%)	CM ^2^ (%)	S/H ^3^	H ^4^ (M)	A/B ^5^	RA ^6^ (%)	LP ^7^ (%)	Ag:B ^8^ (%)	Sp/B ^9^ (%)
MM4	50	50	1	10	0.7	10	10	3	1
MM5	50	50	1	16	0.7	10	10	3	1
MM6	50	50	1	10	0.6	10	10	3	1

^1^ Fly ash to binder content; ^2^ cement to binder ct.; ^3^ sodium sil. to hyd. ratio; ^4^ sodium hyd. concentration; ^5^ alkaline sol. to binder rt.; ^6^ recycled to total aggr. rt.; ^7^ limest. pow. to total aggr rt.; ^8^ aggregates to binder ratio; ^9^ superplast. to binder rt.

**Table 5:** Key material ratios of the third group of mortar mixtures.

Sample	FA ^1^ (%)	CM ^2^ (%)	S/H ^3^	H ^4^ (M)	A/B ^5^	RA ^6^ (%)	LP ^7^ (%)	Ag:B ^8^ (%)	Sp/B ^9^ (%)
MM7	50	50	2	16	0.7	10	0	3	1
MM8	50	50	2	16	0.6	0	10	3	1
MM9	50	50	2	16	0.7	10	10	3	1

^1^ Fly ash to binder content; ^2^ cement to binder ct.; ^3^ sodium sil. to hyd. ratio; ^4^ sod. hyd. concentration; ^5^ alkaline sol. to binder rt.; ^6^ recycled to total aggr. rt.; ^7^ limest. pow. to total aggr rt.; ^8^ aggregates to binder ratio; ^9^ superplast. to binder rt.

**Table 6:** Composition of the reference sample.

Sample	FA ^1^ (g)	C ^2^ (g)	G ^3^ (g)	H ^4^ (g)	S ^5^ (g)	A ^6^ (g)
MM10	281.3	93.8	0.0	100.0	200.0	1012.5

^1^ Fly ash mass in weight; ^2^ cement wt.; ^3^ waste glass wt.; ^4^ sodium hydroxide wt.; ^5^ sodium silicate wt.; ^6^ aggregates wt.

**Table 7:** Variation in the relative weight of the components of the mortars in relation to the reference sample.

Sample	ΔFA ^1^ (%)	ΔC ^2^ (%)	ΔG ^3^ (%)	ΔH ^4^ (%)	ΔS ^5^ (%)	ΔA ^6^ (%)
MM11	−0.56	−0.19	1.0	0	−1.0	0.75
MM12	−2.81	−0.94	4.0	0	−1.0	0.75
MM13	−5.06	−1.69	7.0	0	−1.0	0.75
MM14	−0.75	−0.25	1.0	1.0	−1.0	0
MM15	−3.00	−1.00	4.0	1.0	−1.0	0
MM16	−5.25	−1.75	7.0	1.0	−1.0	0

^1^ Fly ash content variation; ^2^ cement ct. var.; ^3^ waste glass ct. var.; ^4^ sodium hydroxide ct. var.; ^5^ sodium silicate ct. var.; ^6^ aggregates ct. var.

**Table 8:** Key material ratios of the mortar mixtures—first approach.

Sample	FA ^1^ (%)	CM ^2^ (%)	Gl ^3^ (%)	S/H ^4^	H ^5^ (M)	A/B ^6^	RA ^7^ (%)	Ag:B ^8^	T ^9^ (°C)
MM10	75.0	25.0	0	2	10	0.8	10	3	80
MM11	71.4	23.8	1	2	10	0.7	10	3	80
MM12	60.8	20.3	4	2	10	0.7	10	3	80
MM13	50.1	16.7	7	2	10	0.7	10	3	80

^1^ Fly ash to binder content; ^2^ cement to binder ct.; ^3^ glass powder to binder ct. ^4^ sodium sil. to hyd. ratio; ^5^ sodium hyd. concentration; ^6^ alkaline sol. to binder rt.; ^7^ recycled to total aggr. rt.; ^8^ aggregates to binder ratio; ^9^ curing temperature.

**Table 9:** Key material ratios of the mortar mixtures—second approach.

Sample	FA ^1^ (%)	CM ^2^ (%)	Gl ^3^ (%)	S/H ^4^	H ^5^ (M)	A/B ^6^	RA^7^ (%)	Ag:B ^8^	T ^9^ (°C)
MM10	75.0	25.0	0	2	10	0.8	10	3	80
MM14	71.4	23.8	1	2	10	0.8	10	3	80
MM15	60.6	20.2	4	2	10	0.8	10	3	80
MM16	49.8	16.6	7	2	10	0.8	10	3	80

^1^ Fly ash to binder content; ^2^ cement to binder ct.; ^3^ glass powder to binder ct.; ^4^ sodium sil. to hyd. ratio; ^5^ sodium hyd. concentration; ^6^ alkaline sol. to binder rt.; ^7^ recycled to total aggr. rt.; ^8^ aggregates to binder ratio; ^9^ curing temperature.

**Table 10:** The *p*-values of the F-tests performed with the values of the compressive strength for different curing periods.

Curing Period	Compressive Strength
Short-term (7/8 days)	*p*-value = 0.378
Medium-term (14 days)	*p*-value = 0.245
Long-term (28 days)	*p*-value = 0.0309

## Data Availability

Data are contained within the article or are available on request from the corresponding author.
